# Supplementary material for: Regular perturbation on the group-velocity dispersion parameter for nonlinear fibre-optical communications
Source: Nat Commun. 2020 Feb 18;11:933. doi: 10.1038/s41467-020-14503-w (PMC7028946; doi:10.1038/s41467-020-14503-w)
Supplement: Supplementary file 1 — Supplementary Information [file 41467_2020_14503_MOESM1_ESM.pdf]

Supplementary Information

Regular Perturbation on the Group-Velocity Dispersion Parameter for Nonlinear Fibre-Optical Communications

Oliari *et al.*

Supplementary Information

Vinícius Oliari[1], Erik Agrell[2*], and Alex Alvarado[1]

[1]Information and Communication Theory (ICT) Lab, Signal Processing Systems (SPS) Group, Department of Electrical Engineering, Eindhoven University of Technology, 5600 MB Eindhoven, The Netherlands
[2]Department of Electrical Engineering, Chalmers University of Technology, Gothenburg SE-41296, Sweden
[*]agrell@chalmers.se

Supplementary Note 1: Definitions

This document provides the proofs of the three theorems presented in the manuscript. For completeness and for these notes to be self-contained, we will repeat the theorems and the necessary definitions.

The theorems are related to approximated solutions of the normalized nonlinear Schödinger equation

$$\frac{\partial A(t,z)}{\partial z} = -\frac{j\beta_2}{2}\frac{\partial^2 A(t,z)}{\partial t^2} + j\gamma \mathrm{e}^{-\alpha z}|A(t,z)|^2 A(t,z), \quad (1)$$

obtained by regular perturbation theory. Regarding the notation in this document, we define

$$\mathcal{D}_z\{f\}(t) \triangleq (f * h(\cdot,z))(t), \quad (2)$$

$$h(t,z) = \frac{1}{\sqrt{j2\pi\beta_2 z}}\mathrm{e}^{-\frac{j}{2\beta_2 z}t^2}, \quad (3)$$

as the dispersion operator applied to a function f of time, which can be used to obtain the dispersion-only solution ($\gamma = 0$)

$$A_M(t,z) = \mathcal{D}_z\{A(\cdot,0)\}(t) \quad (4)$$

of Supplementary Equation (1). Analogously, the solution for $\beta_2 = 0$, called the nonlinear phase noise (NLPN) model, is given by

$$A_M(t,z) = A(t,0)\mathrm{e}^{j\gamma|A(t,0)|^2 G(z)}, \quad (5)$$

$$G(z) = \int_0^z \mathrm{e}^{-\alpha u}\mathrm{d}u = \frac{1-\mathrm{e}^{-\alpha z}}{\alpha}. \quad (6)$$

In the frequency domain, we define the Fourier transform of a function $A(\cdot,z)$ as

$$\mathcal{F}\{A(\cdot,z)\}(\omega) = \tilde{A}(\omega,z) \triangleq \int_{-\infty}^{\infty} A(t,z)\mathrm{e}^{+j\omega t}\mathrm{d}t \quad (7)$$

which depends on the angular frequency ω and is evaluated at distance z. The inverse Fourier transform of $\tilde{A}(\cdot,z)$ is given by

$$\mathcal{F}^{-1}\{\tilde{A}(\cdot,z)\}(t) = A(t,z) = \frac{1}{2\pi}\int_{-\infty}^{\infty}\tilde{A}(\omega,z)\mathrm{e}^{-j\omega t}\mathrm{d}\omega. \quad (8)$$

Supplementary Note 2: Proof of Theorem 1

In the regular perturbation (RP) on γ, we consider that the solution of Supplementary Equation (1) can be written as an expansion in terms of the nonlinear coefficient γ

$$A(t,z) = \sum_{k=0}^{\infty}\gamma^k A_k(t,z), \quad (9)$$

and the first order RP is obtained by truncating the expansion at $k = 1$.

Theorem 1. *Let A be the solution of the NLSE in Supplementary Equation (1) with initial condition $A(\cdot,0)$. Then, A can be approximated by A_M, the first order RP on the nonlinear coefficient γ of Supplementary Equation (1), written as*

$$A_M(t,z) = A_0(t,z) + \gamma A_1(t,z), \quad (10)$$

where

$$A_0(t,z) = \mathcal{D}_z\{A(\cdot,0)\}(t) \quad (11)$$

is the dispersion-only solution in equation (4) and

$$A_1(t,z) = j\int_0^z \mathrm{e}^{-\alpha u}\mathcal{D}_{z-u}\left\{|A_0(\cdot,u)|^2 A_0(\cdot,u)\right\}(t)\,du. \quad (12)$$

Proof. Substituting Supplementary Equation (9) in Supplementary Equation (1) yields

$$\sum_{k=0}^{\infty}\gamma^k\frac{\partial A_k(t,z)}{\partial z} + \frac{j\beta_2}{2}\sum_{k=0}^{\infty}\gamma^k\frac{\partial^2 A_k(t,z)}{\partial t^2} = j\gamma \mathrm{e}^{-\alpha z}\sum_{m=0}^{\infty}\sum_{n=0}^{\infty}\sum_{p=0}^{\infty}\gamma^{m+n+p}A_m(t,z)A_n^*(t,z)A_p(t,z). \quad (13)$$

Every term in Supplementary Equation (13) is multiplied by a term γ^l with $l = 0, 1, \ldots$. We will solve Supplementary Equation (13) by equating the terms with same power of γ for increasing values of l.

For γ^0 ($l = 0$), we obtain

$$\frac{\partial A_0(t,z)}{\partial z} = -\frac{j\beta_2}{2}\frac{\partial^2 A_0(t,z)}{\partial t^2}, \tag{14}$$

whose solution (with initial condition $A_0(t,0) = A(t,0)$) is

$$A_0(t,z) = \mathcal{D}_z\{A(\cdot,0)\}(t). \tag{15}$$

For γ^1 ($l = 1$), we obtain

$$\frac{\partial A_1(t,z)}{\partial z} = -\frac{j\beta_2}{2}\frac{\partial^2 A_1(t,z)}{\partial t^2} + j\mathrm{e}^{-\alpha z}S(t,z), \tag{16}$$

where

$$S(t,z) = |A_0(t,z)|^2 A_0(t,z). \tag{17}$$

We solve Supplementary Equation (16) in the Fourier domain as

$$\frac{\partial \tilde{A}_1(\omega,z)}{\partial z} = \frac{j\beta_2\omega^2}{2}\tilde{A}_1(\omega,z) + j\mathrm{e}^{-\alpha z}\tilde{S}(\omega,z). \tag{18}$$

Grouping the terms related to A_1, Supplementary Equation (18) is expressed as

$$\frac{\partial\left[\tilde{A}_1(\omega,z)\mathrm{e}^{-\frac{j\beta_2}{2}\omega^2 z}\right]}{\partial z} = j\mathrm{e}^{-\alpha z}\mathrm{e}^{-\frac{j\beta_2}{2}\omega^2 z}\tilde{S}(\omega,z). \tag{19}$$

By using the initial condition $A_1(t,0) = 0$, Supplementary Equation (19) is solved as

$$\tilde{A}_1(\omega,z) = j\int_0^z \mathrm{e}^{-\alpha u}\mathrm{e}^{\frac{j\beta_2}{2}\omega^2(z-u)}\tilde{S}(\omega,u)\mathrm{d}u. \tag{20}$$

The last step in the proof is to take the inverse Fourier transform of Supplementary Equation (20). To do this, we use the fact that the operator $\mathcal{D}_z$ in Supplementary Equation (2) can be represented in the frequency domain as

$$\tilde{\mathcal{D}}_z\{\tilde{f}\}(\omega) = \tilde{f}(\omega)\mathrm{e}^{\frac{j\beta_2}{2}\omega^2 z}. \tag{21}$$

Replacing $\tilde{f}$ by $\tilde{S}(\cdot,u)$ in Supplementary Equation (21) gives

$$\tilde{\mathcal{D}}_z\{\tilde{S}(\cdot,u)\}(\omega) = \tilde{S}(\omega,u)\mathrm{e}^{\frac{j\beta_2}{2}\omega^2 z}. \tag{22}$$

Now, instead of applying the operator $\tilde{D}_z$ at distance z, we use it at distance $z - u$, obtaining

$$\tilde{\mathcal{D}}_{z-u}\{\tilde{S}(\cdot,u)\}(\omega) = \tilde{S}(\omega,u)\mathrm{e}^{\frac{j\beta_2}{2}\omega^2(z-u)}. \tag{23}$$

The time-frequency dual representation of the operator $\mathcal{D}_z$ is

$$\mathcal{F}^{-1}\{\tilde{\mathcal{D}}_{z-u}\{\tilde{f}\}\}(t) = \mathcal{D}_{z-u}\{f\}(t), \tag{24}$$

and thus, the inverse Fourier transform of the left-hand side of Supplementary Equation (23) is

$$\mathcal{F}^{-1}\{\tilde{\mathcal{D}}_{z-u}\{\tilde{S}(\cdot,u)\}\}(t) = \mathcal{D}_{z-u}\{S(\cdot,u)\}(t). \tag{25}$$

Using Supplementary Equation (25) together with Supplementary Equation (20), we have

$$\begin{aligned}
A_1(t,z) &= \mathcal{F}^{-1}\{\tilde{A}_1(\cdot,z)\}(t) \\
&= \mathcal{F}^{-1}\left\{j\int_0^z \mathrm{e}^{-\alpha u}\mathrm{e}^{\frac{j\beta_2}{2}(\cdot)^2(z-u)}\tilde{S}(\cdot,u)\mathrm{d}u\right\}(t) \\
&= j\int_0^z \mathrm{e}^{-\alpha u}\mathcal{F}^{-1}\left\{\mathrm{e}^{\frac{j\beta_2}{2}(\cdot)^2(z-u)}\tilde{S}(\cdot,u)\right\}(t)\mathrm{d}u \\
&= j\int_0^z \mathrm{e}^{-\alpha u}\mathcal{F}^{-1}\left\{\tilde{\mathcal{D}}_{z-u}\{\tilde{S}(\cdot,u)\}\right\}(t)\mathrm{d}u \\
&= j\int_0^z \mathrm{e}^{-\alpha u}\mathcal{D}_{z-u}\left\{|A_0(\cdot,u)|^2 A_0(\cdot,u)\right\}(t)\mathrm{d}u,
\end{aligned} \tag{26}$$

where in the last step we used Supplementary Equation (17). ■

Supplementary Note 3: Proof of Theorem 2

In the RP on β_2, we consider that the solution of Supplementary Equation (1) can be written as an expansion in terms of the nonlinear coefficient β_2

$$A(t,z) = \sum_{k=0}^{\infty}\beta_2^k A_k(t,z). \tag{27}$$

and the first order RP is obtained by truncating the expansion at $k = 1$.

Theorem 2. *Let A be the solution of the NLSE in equation (1) with initial condition $A(\cdot,0)$. Then, A can be approximated by A_M, the first order RP on the linear coefficient β_2 of equation (1), written as*

$$A_M(t,z) = A_0(t,z) + \beta_2 A_1(t,z), \tag{28}$$

where

$$A_0(t,z) = A(t,0)\mathrm{e}^{j\gamma|A(t,0)|^2 G(z)}, \tag{29}$$

and

$$A_1(t,z) = B(t,z)\mathrm{e}^{j\gamma|A(t,0)|^2 G(z)}, \tag{30}$$

with B given by

$$\begin{aligned}
B(t,z) =& -M(t)z + G_1(z)R(t) + G_2(z)P(t) \\
& -2j\gamma A(t,0)\Re\{A^*(t,0)V(t,z)\}, && (31)\\
V(t,z) =& \; G(z)\left[M(t)z - G_1(z)R(t) - G_2(z)P(t)\right] \\
& -G_1(z)M(t) + G_2(z)R(t) + G_3(z)P(t), && (32)
\end{aligned}$$

$$M(t) = \frac{j}{2}\frac{\partial^2 A(t,0)}{\partial t^2}, \tag{33}$$

$$R(t) = \frac{\gamma}{2}A(t,0)\frac{\partial^2 |A(t,0)|^2}{\partial t^2} + \gamma\frac{\partial A(t,0)}{\partial t}\frac{\partial |A(t,0)|^2}{\partial t}, \tag{34}$$

$$P(t) = \frac{j\gamma^2}{2}A(t,0)\left(\frac{\partial |A(t,0)|^2}{\partial t}\right)^2, \tag{35}$$

$$G_1(z) = \frac{\alpha z + \mathrm{e}^{-\alpha z} - 1}{\alpha^2}, \tag{36}$$

$$G_2(z) = \frac{2\alpha z + 4\mathrm{e}^{-\alpha z} - \mathrm{e}^{-2\alpha z} - 3}{2\alpha^3}, \tag{37}$$

$$G_3(z) = \frac{6\alpha z + 18\mathrm{e}^{-\alpha z} - 9\mathrm{e}^{-2\alpha z} + 2\mathrm{e}^{-3\alpha z} - 11}{6\alpha^4}. \tag{38}$$

Proof. To obtain A_0 and A_1 in Supplementary Equation (29) and Supplementary Equation (30), we substitute Supplementary Equation (27) in Supplementary Equation (1), resulting in

$$\sum_{k=0}^{\infty}\beta_2^k\frac{\partial A_k(t,z)}{\partial z} + \frac{j\beta_2}{2}\sum_{k=0}^{\infty}\beta_2^k\frac{\partial^2 A_k(t,z)}{\partial t^2} = j\gamma\mathrm{e}^{-\alpha z}\sum_{m=0}^{\infty}\sum_{n=0}^{\infty}\sum_{p=0}^{\infty}\beta_2^{m+n+p}A_m(t,z)A_n^*(t,z)A_p(t,z). \tag{39}$$

We now follow a procedure analogous to that in Supplementary Note 1. First, we equate the terms that depend on the 0-th power of β_2. This gives

$$\frac{\partial A_0(t,z)}{\partial z} = j\gamma\mathrm{e}^{-\alpha z}|A_0(t,z)|^2 A_0(t,z), \tag{40}$$

which has the same solution as the NLPN model in Supplementary Equation (5). The solution of Supplementary Equation (40) is Supplementary Equation (29), with G given by Supplementary Equation (6) and using the input field as initial condition, i.e., $A_0(t,0) = A(t,0)$.

The next step is to obtain A_1 by equating the terms that depend on β_2^1. This gives

$$\frac{\partial A_1(t,z)}{\partial z} + \frac{j}{2}\frac{\partial^2 A_0(t,z)}{\partial t^2} = j\gamma\mathrm{e}^{-\alpha z}(2|A_0(t,z)|^2 A_1(t,z) + A_1^*(t,z)A_0^2(t,z)). \tag{41}$$

We now claim that Supplementary Equation (41) with the boundary condition $A_1(t,0) = 0$ is solved by

$$A_1(t,z) = B(t,z)\mathrm{e}^{j\gamma|A(t,0)|^2 G(z)}, \tag{42}$$

where

$$B(t,z) = -\int_0^z F(t,u)\mathrm{d}u - 2j\gamma A(t,0)\Re\left\{A^*(t,0)\int_0^z (G(z) - G(u))F(t,u)\mathrm{d}u\right\}, \tag{43}$$

$$F(t,z) = \frac{j}{2}\frac{\partial^2 A_0(t,z)}{\partial t^2}\mathrm{e}^{-j\gamma|A(t,0)|^2 G(z)}. \tag{44}$$

To prove that Supplementary Equations (42)–(44) solve Supplementary Equation (41), let

$$L(t,z) = \mathrm{e}^{j\gamma|A(t,0)|^2 G(z)}. \tag{45}$$

Then, we can rewrite the first term in Supplementary Equation (41) using Supplementary Equation (42), Supplementary Equation (45) and Supplementary Equation (43) as

$$\begin{aligned} T_1 = \frac{\partial A_1(t,z)}{\partial z} &= B(t,z)\frac{\partial L(t,z)}{\partial z} + L(t,z)\frac{\partial B(t,z)}{\partial z} \\ &= j\gamma\mathrm{e}^{-\alpha z}|A(t,0)|^2 B(t,z)L(t,z) - L(t,z)F(t,z) \\ &\quad - 2j\gamma\mathrm{e}^{-\alpha z}A(t,0)L(t,z)\Re\left\{A^*(t,0)\int_0^z F(t,u)\mathrm{d}u\right\}. \end{aligned} \tag{46}$$

The second term in Supplementary Equation (41) follows from Supplementary Equation (44) and Supplementary Equation (45) as

$$T_2 = \frac{j}{2}\frac{\partial^2 A_0(t,z)}{\partial t^2} = L(t,z)F(t,z). \tag{47}$$

For the other two remaining terms, Supplementary Equation (30), Supplementary Equation (42), and Supplementary Equation (45) yield

$$\begin{aligned} T_3 &= 2j\gamma|A_0(t,z)|^2 A_1(t,z)\mathrm{e}^{-\alpha z} \\ &= 2j\gamma|A(t,0)|^2 B(t,z)L(t,z)\mathrm{e}^{-\alpha z}, \end{aligned} \tag{48}$$

$$\begin{aligned} T_4 &= j\gamma A_0^2(t,z)A_1^*(t,z)\mathrm{e}^{-\alpha z} \\ &= j\gamma A^2(t,0)L(t,z)B^*(t,z)\mathrm{e}^{-\alpha z}. \end{aligned} \tag{49}$$

Now, combining the four terms to form Supplementary

Equation (41), we obtain

$$
\begin{aligned}
&-T_1 - T_2 + T_3 + T_4 \\
&\quad = j\gamma \mathrm{e}^{-\alpha z} |A(t,0)|^2 B(t,z) L(t,z) \\
&\qquad + 2j\gamma \mathrm{e}^{-\alpha z} A(t,0) L(t,z) \Re\left\{ A^*(t,0) \int_0^z F(t,u)\mathrm{d}u \right\} \\
&\qquad + j\gamma A^2(t,0) L(t,z) B^*(t,z) \mathrm{e}^{-\alpha z} \\
&\quad = j\gamma \mathrm{e}^{-\alpha z} L(t,z) A(t,0) \left[2\Re\left\{ A^*(t,0) \int_0^z F(t,u)\mathrm{d}u \right\} \right. \\
&\qquad \left. + A^*(t,0) B(t,z) + A(t,0) B^*(t,z) \right] \\
&\quad = j\gamma \mathrm{e}^{-\alpha z} L(t,z) A(t,0) \\
&\qquad \cdot \left[2\Re\left\{ A^*(t,0) \left(\int_0^z F(t,u)\mathrm{d}u + B(t,z) \right) \right\} \right] \\
&\quad = 0, \qquad (50)
\end{aligned}
$$

where the last equation comes from the fact that $A^*(t,0)(\int_0^z F(t,u)\mathrm{d}u + B(t,z))$ is purely imaginary, which follows from using the definition of B in Supplementary Equation (43). This shows that Supplementary Equations (42)–(45) are a solution of Supplementary Equation (41).

Now, we will show that the integrals in Supplementary Equation (43) can be evaluated analytically. By substituting A_0 from Supplementary Equation (29) in the expression for F in Supplementary Equation (44), we obtain

$$
\begin{aligned}
F(t,z) &= \frac{j}{2}\frac{\partial^2 A(t,0)}{\partial t^2} - G(z)\left[\frac{\gamma}{2} A(t,0) \frac{\partial^2 |A(t,0)|^2}{\partial t^2} \right. \\
&\qquad \left. + \gamma \frac{\partial A(t,0)}{\partial t} \frac{\partial |A(t,0)|^2}{\partial t} \right] \\
&\qquad - G^2(z) \left[\frac{j\gamma^2}{2} A(t,0) \left(\frac{\partial |A(t,0)|^2}{\partial t} \right)^2 \right] \\
&= M(t) - G(z)R(t) - G^2(z)P(t), \qquad (51)
\end{aligned}
$$

where M, R and P only depend on $A(\cdot,0)$ and are respectively given by Supplementary Equation (33), Supplementary Equation (34), and Supplementary Equation (35). Now, by denoting

$$G_1(z) = \int_0^z G(u)\mathrm{d}u = \frac{\alpha z + \mathrm{e}^{-\alpha z} - 1}{\alpha^2}, \qquad (52)$$

$$G_2(z) = \int_0^z G^2(u)\mathrm{d}u = \frac{2\alpha z + 4\mathrm{e}^{-\alpha z} - \mathrm{e}^{-2\alpha z} - 3}{2\alpha^3}, \qquad (53)$$

$$
\begin{aligned}
G_3(z) &= \int_0^z G^3(u)\mathrm{d}u \\
&= \frac{6\alpha z + 18\mathrm{e}^{-\alpha z} - 9\mathrm{e}^{-2\alpha z} + 2\mathrm{e}^{-3\alpha z} - 11}{6\alpha^4}, \qquad (54)
\end{aligned}
$$

the function B in Supplementary Equation (43) can be written as

$$
\begin{aligned}
B(t,z) = &-M(t)z + G_1(z)R(t) + G_2(z)P(t) \\
&- 2j\gamma A(t,0)\Re\{A^*(t,0)V(t,z)\}, \qquad (55) \\
V(t,z) = &\; G(z)\left[M(t)z - G_1(z)R(t) - G_2(z)P(t)\right] \\
&- G_1(z)M(t) + G_2(z)R(t) + G_3(z)P(t), \qquad (56)
\end{aligned}
$$

which completes the proof.

■

Supplementary Note 4: Proof of Theorem 3

Theorem 3. *With ideal distributed amplification, the functions A_0 and A_1 in Supplementary Equation* (29) *and Supplementary Equation* (30) *can be written as*

$$A_0(t,z) = A(t,0)\mathrm{e}^{j\gamma|A(t,0)|^2 z}, \qquad (57)$$

$$A_1(t,z) = B(t,z)\mathrm{e}^{j\gamma|A(t,0)|^2 z}, \qquad (58)$$

where

$$
\begin{aligned}
B(t,z) &= -M(t)z + \frac{z^2}{2}R(t) + \frac{z^3}{3}P(t) \\
&- 2j\gamma A(t,0)\Re\left\{ A^*(t,0) \left[\frac{z^2}{2}M(t) - \frac{z^3}{6}R(t) - \frac{z^4}{12}P(t) \right] \right\}. \qquad (59)
\end{aligned}
$$

and where $M(t)$, $R(t)$, and $P(t)$ are given by Supplementary Equation (33)*, Supplementary Equation* (34)*, and Supplementary Equation* (35)*, respectively.*

Proof. For $\alpha = 0$, the effective length G in Supplementary Equation (6) degenerates to

$$\lim_{\alpha \to 0} G(z) = z \qquad (60)$$

and similarly G_1, G_2, and G_3 are given by

$$G_1(z) = \frac{z^2}{2}, \quad G_2(z) = \frac{z^3}{3}, \quad G_3(z) = \frac{z^4}{4}. \qquad (61)$$

Therefore, $A_0(t,z)$ in Supplementary Equation (29) and $A_1(t,z)$ in Supplementary Equation (30) are given by

$$A_0(t,z) = A(t,0)\mathrm{e}^{j\gamma|A(t,0)|^2 z} \qquad (62)$$

$$A_1(t,z) = B(t,z)\mathrm{e}^{j\gamma|A(t,0)|^2 z}. \qquad (63)$$

The proof is completed by using Supplementary Equation (60) and Supplementary Equation (61) in Supplementary Equation (30) and Supplementary Equation (31), which yields Supplementary Equation (59).

■
